# Pharmacokinetic Modeling of Bepotastine for Determination of Optimal Dosage Regimen in Pediatric Patients with Allergic Rhinitis or Urticaria

**DOI:** 10.3390/pharmaceutics16030334

**Published:** 2024-02-27

**Authors:** Sukyong Yoon, Byung Hak Jin, Choon Ok Kim, Kyungsoo Park, Min Soo Park, Dongwoo Chae

**Affiliations:** 1 Department of Pharmacology, Yonsei University College of Medicine, Seoul 03722, Republic of Korea; sy8705@yuhs.ac (S.Y.); kspark@yuhs.ac (K.P.); 2 Clinical Trials Center, Severance Hospital, Yonsei University College of Medicine, Seoul 03722, Republic of Korea; haky1105@yuhs.ac (B.H.J.); delivery98@yuhs.ac (C.O.K.); 3 Department of Clinical Pharmacology, Severance Hospital, Yonsei University College of Medicine, Seoul 03722, Republic of Korea; 4 Department of Pediatrics, Yonsei University College of Medicine, Seoul 03722, Republic of Korea

**Keywords:** bepotastine, pharmacometrics, popPK, PBPK, optimal dosage, pediatric patients

## Abstract

Bepotastine, a second-generation antihistamine for allergic rhinitis and urticaria, is widely used in all age groups but lacks appropriate dosing guidelines for pediatric patients, leading to off-label prescriptions. We conducted this study to propose an optimal dosing regimen for pediatric patients based on population pharmacokinetic (popPK) and physiologically based pharmacokinetic (PBPK) models using data from two previous trials. A popPK model was built using NONMEM software. A one-compartment model with first-order absorption and absorption lag time described our data well, with body weight incorporated as the only covariate. A PBPK model was developed using PK-Sim software version 10, and the model well predicted the drug concentrations obtained from pediatric patients. Furthermore, the final PBPK model showed good concordance with the known properties of bepotastine. Appropriate pediatric doses for different weight and age groups were proposed based on the simulations. Discrepancies in recommended doses from the two models were likely due to the incorporation of age-dependent physiological factors in the PBPK model. In conclusion, our study is the first to suggest an optimal oral dosing regimen of bepotastine in pediatric patients using both approaches. This is expected to foster safer and more productive use of the drug.

## 1. Introduction

**Figure 1:**
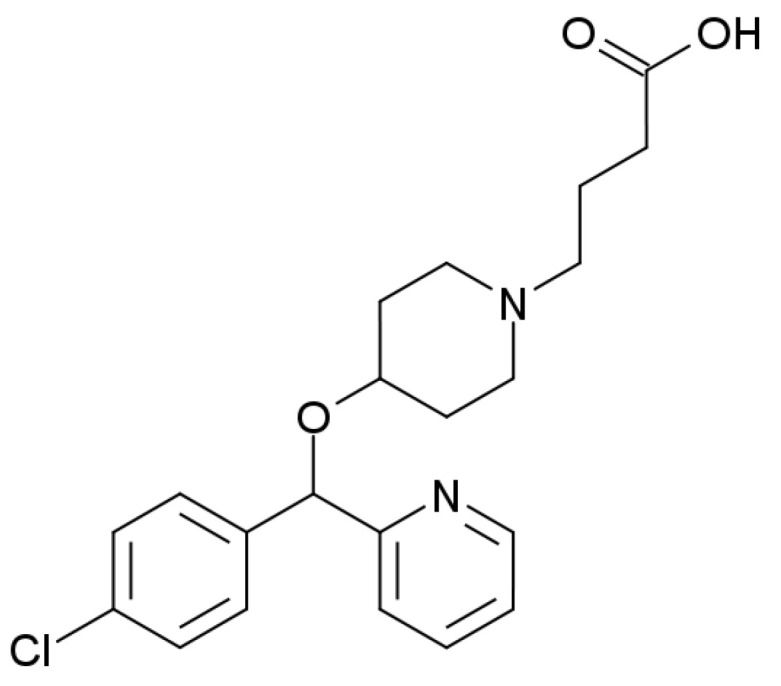
Chemical structure of bepotastine.

Bepotastine (BP) is a second-generation antihistamine prescribed as eye drops or oral tablets. It acts as an inverse agonist of the H_1_-receptors and downregulates allergic inflammation by interfering with histamine action [1]. Like other second-generation drugs, it does not cross the blood-brain barrier and rarely causes central nervous system-related symptoms [1,2]. Moreover, it has a fast onset of action of approximately half an hour [3,4], is excreted primarily through the kidney, and rapidly clears 75–90% of the dose within 24 h [5]. Owing to these advantages, it is widely prescribed to patients of all ages with allergic diseases. The drug is also prescribed as an ophthalmic solution and is contraindicated in patients with hypersensitivity reactions to its ingredients [5]. Some mild adverse events, such as nasopharyngitis, headache, and diarrhea, were reported in clinical trials [5,6]. No drug interactions are reported on the label [5,6]. The chemical structure of BP is presented in Figure 1.

Allergic rhinitis and urticaria are common diseases that affect the quality of life and mental health of pediatric patients [7,8]. Despite its frequent clinical use, no established dosing guidelines exist for pediatric patients. The drug is most often prescribed off-label in clinical practice, unlike in adults who are dosed in accordance with the report [6]. A Japanese study evaluated the efficacy and safety of adult doses in pediatric patients with allergic rhinitis aged 7–15 years given the adult dose and reported no adverse drug reactions [9]. This led to the increased use of BP tablets in children, previously approved and prescribed for adults with allergic rhinitis and urticaria [6]. However, a twofold higher drug exposure was expected in children below seven years of age who were administered 10 mg twice daily [6]. Although no severe adverse events were found, BP is officially indicated only for children aged 7–15 years based on a similar drug exposure to healthy adults. This suggests that the potential risk of overdosing remains a concern in children younger than seven. Data are therefore required in order to determine the proper dosage for children younger than seven.

Conducting large-scale clinical trials in pediatric patients is complex, and pharmacokinetic (PK) modeling and simulation are increasingly being utilized as alternative methods to determine an appropriate dosing regimen [10,11,12,13,14]. Population PK (popPK) and physiologically based pharmacokinetic (PBPK) modeling are two widely used approaches, each with different strengths and weaknesses [10,11,12,13,14]. PopPK modeling is primarily driven by data and is relatively easy. However, extrapolation across species, age, sex, and medical conditions is difficult unless these factors are adequately addressed in the model-building stage. PBPK modeling, on the other hand, offers a potential solution to this problem because its predictions are firmly grounded in physiology [15]. The main drawbacks are the technical difficulty and longer time requirements for building the model, mainly owing to its heavy dependence on previously known drug-related and physiological parameters. Nevertheless, the situation is slowly changing with the development of various PBPK modeling platforms that provide reference databases for physiological parameter values. As such, there is increasing acceptance of using PBPK modeling to predict drug exposure in pediatric patients and adjust doses based on age and body weight [13]. From this perspective, we aimed to utilize both a popPK model that can adequately explain the population and a PBPK model to offer supplementary information regarding our pediatric patients, especially when there is insufficient physiological covariate data, to suggest a more reliable dose recommendation.

Despite the broader adoption of popPK and PBPK modeling to support pediatric drug dosing, no modeling work has been reported for BP. In this study, we aimed to develop popPK and PBPK models to determine an appropriate BP dose for pediatric patients.

## 2. Materials and Methods

### 2.1. Data Collection

Data were obtained from two prospective studies conducted at Severance Hospital (Seoul, Republic of Korea). One was an open-label, single-arm study to evaluate the safety and PK characteristics of pediatric patients aged 2–6 years, and the other was in healthy adults. These studies were approved by the Institutional Review Board of Severance Hospital, and performed in compliance with the Declaration of Helsinki. Thirty pediatric patients were administered 1.5 mL of BP dry syrup (2 mg/mL) twice a day for at least four days, while 32 healthy adults were administered a single dose of 10 mg BP tablets. BP concentration measurements obtained from all participants were used for the PK analysis. A sparse sampling scheme with two samples per pediatric patient was used to measure at different times: one at 0.5 h to 2 h post-dose and the other at 6–12 h post-dose. A total of 13 samples per healthy adult were measured for 24 h at pre-determined time points (pre-dose (0 h), 0.33, 0.67, 1, 1.33, 1.67, 2, 3, 4, 6, 8, 12, and 24 h). Among them, two samples obtained at pre-dose (0 h) and 24 h were excluded from analysis, as their values were below the limit of quantification (BLQ). To measure the plasma concentration of BP, peripheral venous blood was collected in a blood collection tube containing heparin and centrifuged (3000× *g* rpm, 4 °C, 10 min) within 30 min after collection. After centrifugation, only the plasma in the supernatant layer was separated and stored in the freezer at −70 °C until analysis. The plasma concentrations of BP were measured by liquid chromatography-tandem mass spectrometry using an Agilent 6460 Triple Quad mass spectrometer (Agilent Technologies, Santa Clara, CA, USA) in the positive electrospray ionization (ESI+) mode. The calibration curve was linear over the range of 1–1000 ng/mL (coefficient of variation%: 0.1–1.3, accuracy%: 94.3–106.0) with the lower limit of quantification as 1 ng/mL. Information on age, weight, serum creatinine level, estimated glomerular filtration rate (eGFR), and gender were also collected as potential covariates. The demographics of the two studies are summarized in Table 1.

### 2.2. PopPK Model Development

One- and two-compartment models with zero- or first-order absorption, with or without absorption delay, were fitted to the pooled analysis datasets from the two studies. The interindividual variability (IIV) of the PK parameters was assumed to be log-normally distributed. Additive, proportional, and combined models were tested for the residual variability model. Empirical Bayes estimates for individual PK parameters were used to explore the correlations with potential covariates. Body weight was incorporated into allometric equations for clearance (CL) and volume of distribution (V) as follows [16]:

$$Individual\;CL=Typical\;value\;of\;CL\times {(Body\;weight/70)}^{0.75}$$

Individual V=Typical value of V×(Body weight/70)

Age, serum creatinine level, eGFR, and gender were tested as potential covariates of CL and V. Based on the likelihood ratio test, stepwise covariate model building was conducted with *p* < 0.05 (ΔOFV > 3.84) for forward selection and *p* < 0.01 (ΔOFV > 6.63) for backward elimination, and a linear or an exponential model was adopted to describe covariate-parameter relationships. PK analyses were conducted using NONMEM software (version 7.5; ICON Development Solutions, Dublin, Ireland).

### 2.3. PopPK Model Evaluation

The models were evaluated with goodness-of-fit plots, including observation versus population prediction (PRED), observation versus individual prediction (IPRED), conditional weighted residual (CWRES) versus PRED, and CWRES versus time after dose. A visual predictive check (VPC) with 1000 simulated datasets was performed to evaluate the adequacy of the final model. The 5%, 50%, and 95% percentiles of observations were compared with the 90% prediction intervals of the corresponding percentiles to determine the concordance between observed and predicted values.

### 2.4. PopPK Model Simulation

The final PK model was used to determine the optimal dose in pediatric patients. The optimization goal was to achieve similar maximum concentration (C_max_) and area under the concentration-time curve (AUC_last_) values to 10 mg BP administered to healthy adults weighing 70 kg. To achieve this, we generated an integer simulation grid of body weight ranging between 10 and 31 kg, based on the known body weight distribution in children aged 2 to 6 years. If significant covariates other than body weight were identified at the modeling stage, a simulation population was generated by bootstrapping from the pediatric analysis dataset. Deterministic simulations of the final PopPK model were conducted using the typical PK parameter values and the selected covariates. The doses were simulated at intervals of 0.1 mg for each virtual patient.

### 2.5. PBPK Model Development

**Figure 2:**
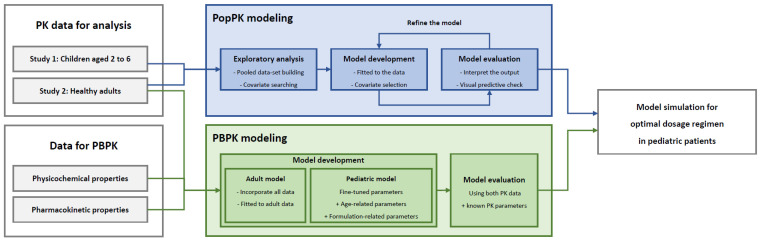
PK modeling workflow for optimal dosage regimen in pediatric patients.

The PBPK model was developed using the same data used to develop the empirical population PK model. The overall workflow was similar to that used in previous studies [13,14] and is presented schematically in Figure 2. First, an adult PBPK model was built and verified using the concentrations observed in the adult data. Subsequently, a pediatric PBPK model was developed by adjusting the physiological values of population-related parameters from those of adults. Then, to integrate the differences in formulations into the model, we applied the Weibull function, which is suitable for describing absorption across various formulations [17].

The physicochemical properties of the drug, such as molecular weight, LogP, pKa, fraction unbound, and water solubility, were obtained from published data, including DrugBank (DB04890). Since no experimental values were available for the parameters related to drug absorption, such as intestinal solubility and permeability, these were calculated based on the physicochemical parameter values and physiological characteristics of the population or assumed based on previous study [18,19,20]. P-glycoprotein (P-gp) was incorporated into the model because it affects drug distribution and prevents drug penetration into the brain [2]. This drug is minimally metabolized by Cytochrome P450 (CYP) isozymes [5] and does not inhibit or induce CYP enzymes. About 75–90% of the drug is excreted unchanged in urine [5]; a minor portion is excreted in bile [21]. Based on the ADME properties of BP mentioned above, PBPK model development was performed using PK-Sim (version 10; Open-systems-pharmacology) software. The database included in the software was used to set typical physiological parameter values of the target population. The optimization and fine-tuning of the relevant parameters, including intestinal permeability and tubular secretion, were attempted using curve-fitting observations. The parameter identification provided by PK-Sim was also utilized to optimize the parameter values associated with Weibull absorption and P-gp. The ages and weights of the children were set equal to those in the pediatric dataset.

### 2.6. PBPK Model Evaluation

A 90% interval of the predicted concentrations obtained from the simulated population generated by PK-Sim was compared with that of the observed concentrations. In addition, we verified whether PK parameters such as C_max_, time to reach maximum concentration (T_max_), and half-life predicted from the developed model were similar to those from previous studies [5,22] and were consistent with the known properties of drugs, including the urinary drug excretion ratio for 24 h. The PK data of Japanese children aged 7 to 15 who received the same tablet dose (10 mg) as adults were also compared with the simulation results of the developed PBPK model for external validation [6]. In clinical trials for children, the drug was administered after a meal, so a delayed gastric emptying time was applied to children based on the literature [6,23].

### 2.7. PBPK Model Simulation

A virtual pediatric patient population aged 2 to 6 years was generated based on the Japanese database provided in the PK-Sim software and the available weight range of Korean children by age group [24]. The optimized parameter values of the final PBPK model were used in the simulation. The distributions of C_max_ and AUC_last_ were estimated for each age group based on 1000 simulated children dosed with 3 mg. The optimal suggested dose was rounded to a single decimal point for practical purposes. The optimal dose was determined based on the AUC_last_ value obtained from the final adult PBPK model, such that the extent of drug exposure to the recommended dose in the pediatric population was similar to that of the standard dose of 10 mg in adults.

## 3. Results

### 3.1. PopPK Model

**Figure 3:**
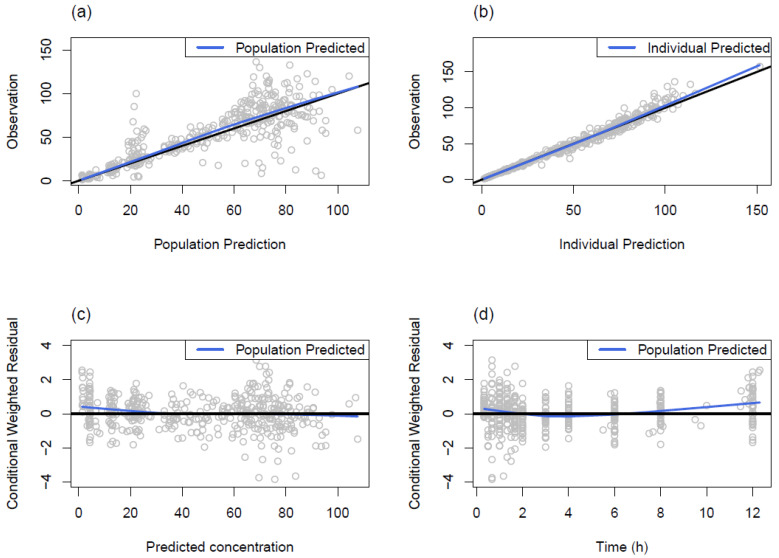
Goodness-of-fit plots of final popPK model. (**a**,**b**) black line: identity line; blue line: smooth, (**c**,**d**) black line: zero residual line; blue line: smooth.

**Figure 4:**
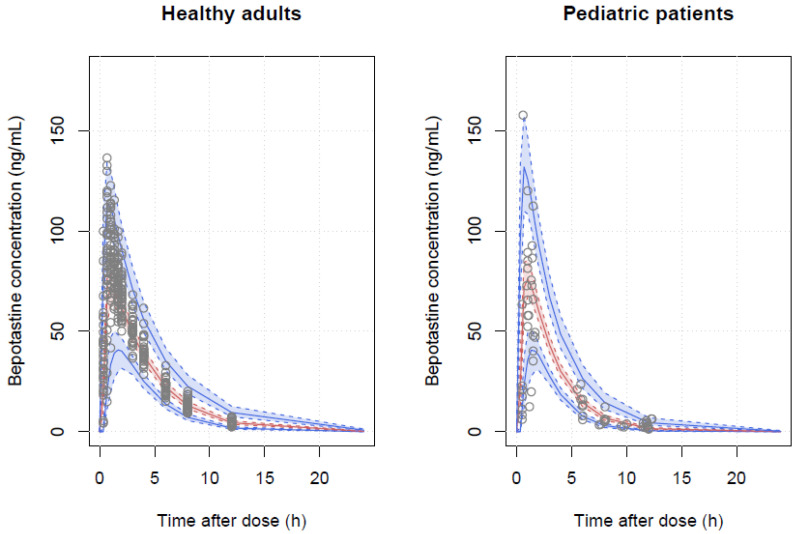
VPC plots of the final popPK model. Grey dots are observations. The red and blue solid lines represent the median, 5% and 95% predicted values, respectively, and the red and blue shades represent their 90% confidence intervals.

A one-compartment model with first-order absorption and absorption lag times was sufficient to describe the data. Individual differences in the absorption rate constant (KA), absorption lag time (ALAG), CL, and V were assumed to be log-normally distributed, and covariances were estimated for CL-V and CL-ALAG pairs. A combined error model was adopted to describe the residual variability, with the additive error variance fixed at 0.1 ng/mL, due to numerical difficulties in estimation. Incorporating body weight into the model resulted in a significant decrease in OFV (*p* < 10^−16^). During covariate model building, serum creatinine was selected in the forward selection process but removed in the backward deletion step such that no covariates were incorporated into the final model. The population estimates of KA, CL, V, and lag time (ALAG) were 4.21 h^−1^, 28.0 L/h, 103.0 L, and 0.27 h, respectively, and all parameters showed reasonable precision with the relative standard error (RSE) below 30%. Details of the final parameter estimates obtained using the first-order conditional estimation (FOCE) method are presented in Table 2. The goodness-of-fit plots of the final PK model are presented in Figure 3 and show that the PK profiles of the observed and predicted concentrations were well superimposed, with no discernible trends. The VPC plot is illustrated in Figure 4, which demonstrates that roughly 90% of the observations were included in the 90% prediction interval.

### 3.2. PopPK Model Simulation

In the final PK model, the inter-individual variability in BP concentrations was well explained by body weight alone in children aged 2–6 years and adults. Considering the weight distribution of Korean children aged 2–6 years, simulations were performed for a total of 22 body weight values ranging between 10 and 31 kg [24]. Optimal doses increased with body weight, and AUC_last_-based doses were consistently higher in all sub-populations compared with C_max_-based doses. The appropriate doses for different body weights are presented in Table 3.

### 3.3. PBPK Model

The adult PBPK model was developed using known physicochemical and PK information. The PK-related parameter values were fine-tuned to improve the fit between the model predictions and observations. First, intestinal permeability calculated from known LogP values showed poor absorption after oral administration. So, the high absorption fraction (F_a_) and high bioavailability of BP could not be explained by those values. To solve this problem, intestinal permeability was optimized based on the information on drugs with a high F_a_ [20]. The turnover number (K_cat_) of P-gp was optimized using a known value from a previous study [2]. Similarly, the parameters related to renal tubular secretion were optimized by curve fitting to the observed data to determine their most likely value because glomerular filtration rate (GFR) alone could not account for the known extent of renal elimination and there was no information on the parameter related to renal secretion in the previous studies [5]. Weibull parameter values, which were determined through data fitting, were also used to explain formulation differences. The parameters used to construct the PBPK model are summarized in Table 4.

**Figure 5:**
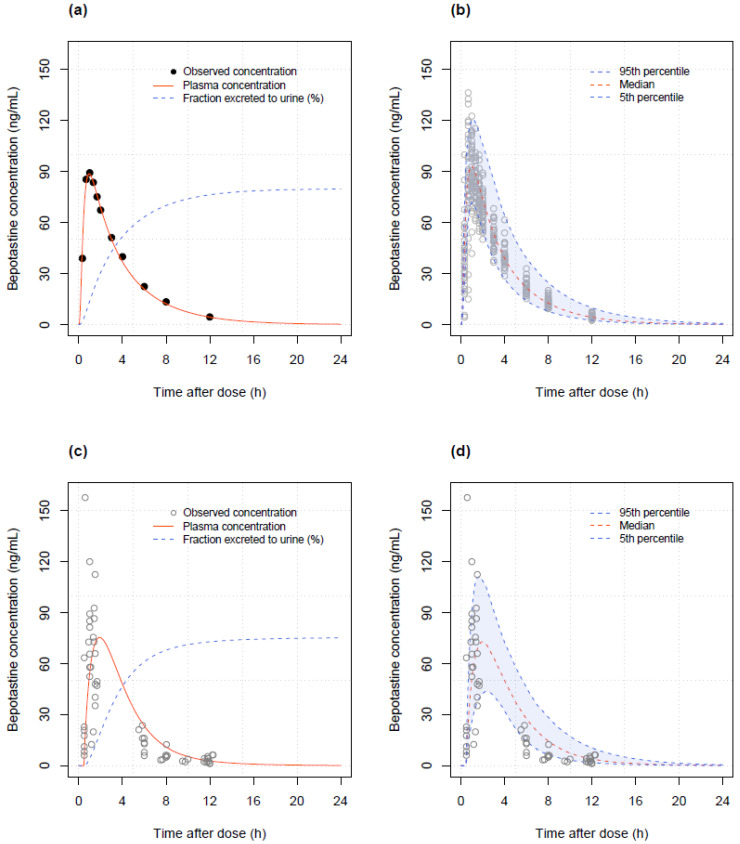
The final (**a**,**b**) adult and (**c**,**d**) pediatric PBPK model. (**a**) black dots: the mean concentration of observation by time, (**b**–**d**) grey dots: observations, (**b**,**d**) red dashed line: the median of the predicted observations; blue dashed line: 90% intervals of prediction.

The final adult PBPK model explained the average value and distribution of the time-concentration profile well and predicted approximately 80% of the 24-h fraction excreted in the urine. The PK parameters, C_max_, AUC_last_, half-life, and T_max_ estimated in the final adult PBPK model were 88.67 ng/mL, 374.65 h∙ng/mL, 2.69 h, and 0.95 h, which were similar to the reported values [26]. In the adult PBPK model with a 10 mg tablet applied, nearly all of the drug was absorbed in the intestine, and the bioavailability was calculated to be 93%. The final pediatric PBPK model well described our data with approximately 90% of the observations included in the 90% prediction interval. The C_max_, AUC_last_, estimated in the final pediatric PBPK model were 75.46 ng/mL, 350.63 h∙ng/mL given a 3 mg dry syrup, slightly lower than those estimated in adults administered 10 mg tablet. The half-life and T_max_ were 4.08 h and 1.90 h, respectively, which were similar to those in adults. In the pediatric PBPK model, the bioavailability was estimated to be 95% and almost all medications were absorbed in the intestine as in adults. The predictions of the final adult and pediatric PBPK models are shown in Figure 5.

### 3.4. PBPK Model Simulation

**Figure 6:**
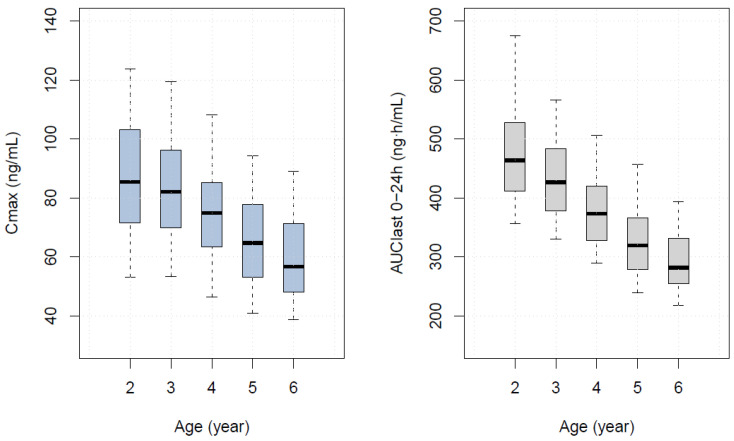
Simulated PK parameters for different age groups. The line within the box indicates the median, and the lower and upper boundaries of the box indicate the 25th and 75th percentiles. Whiskers below and above the box indicate the 10th and 90th percentiles.

**Figure 7:**
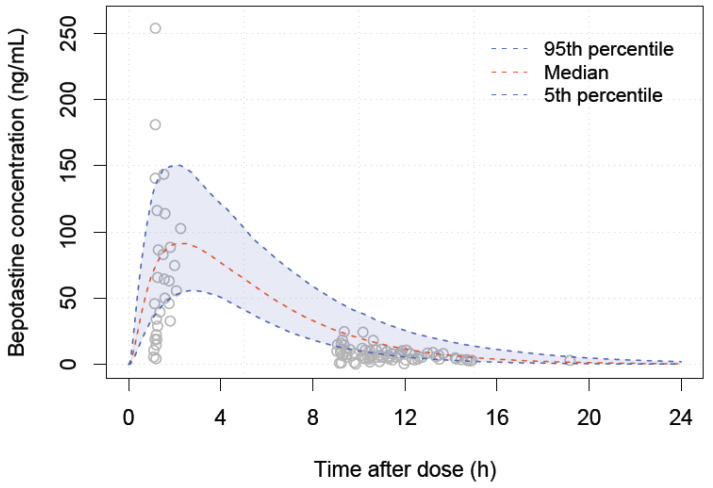
The external validation of PBPK model; grey dots: observations of children aged 7 to 15 years, blue dashed line: 90% intervals of prediction.

The values of C_max_ and AUC_last_ at 3 mg in children aged 2–6 years are shown in Table 5, and the distributions of the two parameters are shown in Figure 6. The exposure expected in a typical 4-year-old group, most similar to that of an adult administered 10 mg, was 3 mg. The appropriate doses in fed state for children aged 2–6 years and their corresponding median body weights are listed in Table 6. The C_max_-based optimal doses predicted by the PBPK model were higher than those predicted by the empirical population PK model. The simulation results for external validation are shown in Figure 7. The predicted 90% concentration interval after administration of 10 mg tablets to 1000 children aged 7 to 15 years is shown along with the observed values. Despite the fact that observations were taken using a digitizer, the predicted concentration range adequately described the data; nonetheless, observations showed an absorption delay of about an hour after administration, which our model could not explain.

### 3.5. Comparison of Predictive Performance of Two Models

Based on the observations (OBS) from the clinical trial in pediatric patients aged 2–6 years and the predicted concentrations (PRED) from the two developed models, the prediction performance was compared based on the following equation (MSE, mean squared error):

$$Prediction\;error=\sum_{i=1}^{n}{\left({OBS}_{i}-{PRED}_{i}\right)}^{2}/n$$

For each model, the prediction errors of the 60 sample points are presented as descriptive statistics. To assess the possible differences in the prediction errors between the absorption and the elimination phases, the samples were split into two periods based on the sampling intervals: those at 0.5 h to 2 h post-dose and the others at 6 to 12 h post-dose. There were no significant differences in prediction errors between the two models. The results are summarized in Table 7.

## 4. Discussion

In this study, we successfully developed pharmacokinetic models of BP for dose optimization in pediatric patients aged 2 to 6 years. Based on model simulations, appropriate doses ranging from 2 to 5 mg were proposed for different age and body weight groups. Our study is the first to derive optimal BP dosing regimens based on the popPK and PBPK models. The dose optimization procedure aimed to minimize the difference between the predicted drug exposure in pediatric patients and that in a typical 70 kg adult patient administered a standard dosing regimen of 10 mg twice daily.

This study used phase 1 clinical trial data from adults to supplement the sparsely sampled pediatric patient concentration data. Covariate analysis based on the popPK model revealed that body weight was the only significant factor affecting CL and V. Overall, the models predicted pediatric data reasonably well. The proportion of observations in pediatric patients outside the 90% prediction interval was approximately 10%, as expected (Figure 4).

Our models also successfully demonstrated a likely safety margin associated with BP exposure. Applying the developed PK model to the results of a previously reported Japanese study involving children aged 7–15 years predicted that a drug exposure of approximately 1.6 times that expected in adults, given the standard dose, would be safe [9]. The simulation results with 1000 replicates of 30 virtual pediatric patients with body weight distribution similar to that previously reported study showed that the pediatric–adult geometric mean ratios of C_max_ and AUC_last_ were 1.92 and 1.67 [9]. Although care must be taken when extrapolating these findings to children aged 2–6 years, this analysis provides an approximate estimate of the maximum exposure associated with no significant adverse events.

PBPK models are increasingly advocated for predicting optimal doses in special populations during drug development [13,14]. However, an important limiting factor is a requirement for sufficient data related to physiological and drug parameters. Fine-tuning of the model parameters is still required to achieve acceptable predictive performance. Considering the reported solubility, permeability, and elimination route of BP, it is presumed to be a BDDCS (Biopharmaceutical Drug Disposition and Classification System) Class 1, but no information was available regarding its classification. Accordingly, the core challenges in developing a PBPK model of BP are the lack of published quantitative data related to the drug compound and the estimation of some parameters, such as intestinal permeability, which require fitting to available concentration–time data. The theoretical intestinal permeability calculated from known LogP values cannot adequately explain drug absorption into the body after oral administration. Although the reason for the significant deviation in the estimated intestinal permeability from the theoretical value is unclear, a possible cause is high variability in permeability in different parts of the intestine. BP is absorbed primarily in the upper part of the small intestine, which could be one of the reasons for the low intestinal permeability observed in previous studies [2,27]. Similarly, owing to the limited availability of quantitative data, tubular secretion values were acquired by fitting the data and considering values from previous studies [5,21]. The tubular secretion rate of 1.33 L/min in the adult PBPK model was adjusted to 0.67 L/min in the pediatric PBPK model based on the available range of renal blood flow in children aged 2–6 years [28,29]. Despite the difficulties, we attempted to minimize the parameter optimization whenever possible. The final PBPK model generated predictions comparable to the popPK model (Figure 5). In addition, simulations were used to present the predicted distribution of the PK parameters in different pediatric age groups when 3 mg of the drug was administered. Thus, the PBPK model offers an alternative dosing regimen that includes age, which is not included as a covariate in the popPK model.

Comparing the predictive performances of the developed popPK and PBPK models, they showed similar prediction errors overall. The popPK model had smaller elimination period prediction errors than the PBPK model, but the differences were not statistically significant. This was an expected result, given that the popPK model was developed by fitting to the dataset. In contrast, the PBPK model parameters were mainly derived from values reported in the literature. Differences in the proposed dosing regimen inevitably accompanied the difference in predictions between the two models. Although there was little difference between the AUC_last_-based doses in the two models, the C_max_-based doses from the PBPK model were higher in all age groups (Table 6). This was likely due to the incorporation of age-dependent physiological factors, such as renal blood flow, into the PBPK model, not accounted for in the popPK model [13,15]. Furthermore, in the PBPK model, gastric emptying time had an effect on the PK profile. As gastric emptying time increased, AUC_last_ exhibited little difference, whereas C_max_ decreased by about 30%, which was similar to cetirizine, one of the second-generation antihistamines [5,30]. Given this, the dose recommended by the PBPK model for each age group based on C_max_ is about 10% greater than the dose suggested by the popPK model in the fasting state, decreasing the difference in the recommended dose. To reconcile the different dose proposals based on the two models, we suggest that for children aged two years, accounting for approximately 10% of the total pediatric data, the dose proposed based on the PBPK model might be more appropriate, given the prediction error of the PBPK model was 427.0, smaller than 1730.4 of the popPK model. This concurs with the widely acknowledged fact that there is rapid maturation of organ systems up to 2 years of age and that weight alone would be insufficient to account for PK differences. Hence, our results suggest that the PBPK model would be helpful when lower doses are administered to patients under three years of age. In this regard, the two models can be used complementarily, depending on the clinical situation. However, further studies are needed, as the efficacy and safety of the drug must be considered to establish the optimal dose.

There are some important limitations of our study. Due to the design of the phase 1 clinical trial involving healthy participants, it was not feasible to evaluate physiological factors influencing PK characteristics. All laboratory test results fell within the normal range and did not exhibit any significant correlation with individual PK parameters. Renal maturation function was also tried, but not applicable to our model [31,32]. Additionally, the population/formulation had a significant effect on the absorption rate constant. However, aliasing of the two factors precluded disentangling the contribution of one from the other. Hence, to elucidate the formulation effect on the absorption differences, a follow-up study is required wherein two formulations are administered to the same population.

## 5. Conclusions

Empirical population PK and PBPK models were developed to determine the optimal dose of BP in pediatric patients aged 2–6 years. Despite the minor discrepancies, both models explained the data reasonably well. Body weight seems to be the primary factor generating PK variability; however, age might be essential for consideration in 2-year-old patients. Based on the developed models, doses ranging from 2 to 5 mg were recommended based on age and body weight. This study is expected to serve as a basis for the model-based dose optimization of drugs without established pediatric dosing guidelines.

## Figures and Tables

**Table 1:** Demographics of the two studies included in the analysis.

	Study 1 (Adults, *n* = 32)		Study 2 (Children, *n* = 30)	
Variables	Mean (SD)	Median (Min–Max)	Mean (SD)	Median (Min–Max)
Age (years)	24.97 (4.02)	24 (19–37)	4.33 (1.24)	5 (2–6)
Weight (kg)	70.95 (8.04)	70.8 (56.8–89.6)	18.90 (3.78)	17.8 (13–26)
Creatinine (mg/dL)	0.87 (0.10)	0.85 (0.69–1.10)	0.38 (0.07)	0.36 (0.28–0.52)
eGFR (mL/min/1.73 m^2^)	109.3 (13.30)	112.0 (80–133)	119.4 (16.15)	121.5 (90–152)
Gender (*n*,%)	Male (32, 100)	Female (0, 0)	Male (19, 63)	Female (11, 37)

SD, standard deviation; Min, minimum; Max, Maximum.

**Table 2:** Parameter estimates of the final popPK model.

Structural Parameters		Inter-Individual Variability (IIV)		
Parameter	Estimate (%RSE)	Parameter	Estimate (%RSE)	Shrinkage (%)
KA (h^−1^)	4.21 (5)	IIV of KA (CV%)	112.7 (13)	17.2
CL (L/h)	28.0 (4)	IIV of CL (CV%)	22.5 (12)	14.5
V (L)	103.0 (5)	IIV of V (CV%)	22.0 (21)	17.8
ALAG (h)	0.27 (5)	IIV of ALAG	32.4 (20)	21.3
Exponent of CL	0.75 fixed	CORR of CL-V	0.65 (25)	
Exponent of V	1.0 fixed	CORR of CL-ALAG	0.47 (18)	
Residual variability				
Proportional error	10.3 (9)	Additive error	0.1 fixed	

KA, absorption rate constant; CL, clearance; V, volume of distribution; ALAG, absorption lag time; RSE, relative standard error; CORR, correlation.

**Table 3:** The calculated optimal doses (mg) in children aged 2 to 6 by body weight.

Body Weight (kg)		10	11	12	13	14	15	16	17	18	19	20
Recommended dose based on	C_max_	1.6	1.7	1.9	2.0	2.1	2.3	2.4	2.6	2.7	2.9	3.0
	AUC_last_	2.3	2.5	2.7	2.8	3.0	3.1	3.3	3.5	3.6	3.8	3.9
Body weight (kg)		21	22	23	24	25	26	27	28	29	30	31
Recommended dose based on	C_max_	3.2	3.3	3.4	3.6	3.7	3.9	4.0	4.1	4.3	4.4	4.6
	AUC_last_	4.0	4.2	4.3	4.5	4.6	4.8	4.9	5.0	5.2	5.3	5.4

**Table 4:** Input parameters for PBPK model.

Parameter	Initial Value	Final Values	References
Physico-chemical properties			
Molecular weight (g/mol)	388.88		DrugBank
LogP	0.55		Chemaxon (in silico)
pKa	4.10/9.39		Chemaxon (in silico)
Fraction unbound	0.45		Talion label [6], NIH (NCATS)
Water Solubility (mg/mL)	40.2		Paper (in vitro) [25]
Pharmacokinetic properties			
Absorption			
Intestinal permeability (cm/s)	5.0 × 10^−4^	3.04 × 10^−4^	Paper (in vitro/in vivo) [20], fitted to data
Weibull absorption		(Tablet, Dry syrup)	Parameter identification by PK-Sim
Dissolution time (min)		85, 32.63	
Lag time (min)		0, 28.40	
Dissolution shape		0.84, 0.55	
Distribution			
P-gp (ABCB1), Km (umol/L)	1.25		Paper (in vivo) [2]
P-gp (ABCB1), Kcat (1/s)	6.47	5.41	Paper (in vivo) [2] Parameter identification by PK-Sim
Blood to plasma ratio ^†^	0.69		Calculated by PK-Sim
Partition coefficient ^†^	0.32		Calculated by PK-Sim
Elimination			
Tubular secretion (L/min)	0.7	1.33/* 0.67	Fitted to data
CYP-related parameters	None		DrugBank, Bepreve label [5]

* 0.67 were used for children. ^†^ The values were calculated by the PK-Sim standard distribution model.

**Table 5:** Summary of simulated PK parameters stratified by age-group.

	PK Parameters	
Age (Year)	AUC_last_ (H·ng/mL) (5th Percentile–95th Percentile)	C_max_ (ng/mL) (5th Percentile–95th Percentile)
2	463.67 (355.96–675.47)	85.46 (53.11–123.77)
3	426.64 (329.97–566.67)	82.08 (53.40–119.54)
4	373.38 (289.31–506.73)	74.88 (46.50–108.20)
5	319.45 (239.37–456.62)	64.70 (40.80–94.42)
6	281.81 (217.15–393.55)	56.68 (38.84–88.98)

Values are presented as median.

**Table 6:** Recommended doses (mg) estimated from the two models.

Age (Year)	* Weight (kg) Median (5th Percentile–95th Percentile)	Based on AUC_last_		Based on C_max_	
		PBPK	PopPK ^†^	PBPK	PopPK ^†^
2	14.0 (13.1–15.4)	2.4	3.0	3.1	2.1
3	14.4 (13.4–16.4)	2.7	3.0	3.3	2.2
4	15.9 (14.2–18.6)	3.0	3.3	3.5	2.4
5	17.9 (15.8–20.8)	3.5	3.6	4.1	2.7
6	19.6 (17.0–23.2)	4.0	3.8	4.7	3.0

* The weight of each age group was estimated by a simulated population. ^†^ The recommended dose for the age group estimated by PopPK considered only the median weight.

**Table 7:** Relative prediction error of two models.

	Number of Samples	PopPK	PBPK	^†^ *p*-Value
Prediction error	60 (Total)	750.6 ± 1574.8	725.0 ± 2533.1	0.95
	30 (Absorption period)	1489.0 ± 1979.1	1427.8 ± 3468.6	0.93
	30 (Elimination period)	12.2 ± 17.5	22.0 ± 39.3	0.22

Values are presented as the mean squared error (MSE) ± standard deviation. ^†^ *p*-values were calculated by *t*-test.

## Data Availability

The data supporting reported results are available from the corresponding author upon approval of a written request by Severance Hospital. The data are not publicly available because of confidentiality.
